# Masso-Therapeutics

**Published:** 1889-06

**Authors:** 


*Masso-Therapeutics.* Fourth Edition. By WILLIAM MURRELL, M.D. Pp. 236. London: H. K. LEWIS. 1889.

*Lectures on Massage and Electricity.* By THOMAS STRETCH DOWSE, M.D. Pp. 379. Bristol: JOHN WRIGHT AND CO. *No date.*

These two works, dealing with the same subject of Massage, may properly be reviewed together.

The objects of these works may partly be deduced from their prefaces.

Dr. Murrell says: "By masso-therapeutics I mean the scientific aspect of the subject; massage, that is, simply as a therapeutic agent, and not massage as a means of earning a living or as a modified form of hotel-keeping. It must be admitted that many people regard massage and all that appertains to it with a good deal of suspicion, and not without reason." Note that Dr. Tibbits has published a set of lectures specially designed to teach persons how to earn a living by massage.

Dr. Stretch Dowse hopes, with respect to his lecturse, "that in their publication his worst enemies will see no reason to insinuate that they have been produced from any other than purely professional motives; . . . and he lays no claim to any special merit or originality, neither has he brought forward any *wonderful* cases that he has cured." We heartily re-echo his hope; but we are sorry that he has not given us some wonderful cases of cure. Surely they would have enhanced our estimate of the value of massage. Most men do record their best cases in support of a line of treatment they advocate. Why should not our author?

In each of these works a special page or pages of advertisement of "works by the same author" is provided for our perusal.

In each of them we are warned again and again against the tendency which massage has to degenerate into quackery. In each of them we have impressed upon us the extensive and prolonged education which the massage practitioner must undergo. Says Dr. Murrell: "There is as much difference between Metzger's massage and the so-called English massage as there is between champagne and gooseberry. It is oysters and marcobrunner *versus* ginger-beer and whelks." This, it must be noted, is the work which specially claims to deal with the scientific aspect of massage. And again: "It is just as difficult to learn massage in a few easy lessons as it is to become a *prima donna* by this simple means."

Dr. Stretch Dowse comes near our homes. He tells us (p. 253) that 'Mr. W. H. Freeman, the Surgeon" (note the definite article) "to the Bath United Hospital, has written an intensely interesting work entitled 'Thermal Baths of Bath, with the Aix Massage and Natural Vapour Treatment.' Mr. Freeman did me the honour," &c., &c. . . "Nothing can surpass the new baths which have been and are still being erected under the very able supervision of Mr. Freeman." We wonder how Mr. Freeman and his colleagues will like this.

We have preferred to let these authors review themselves as far as our space will permit. We wish we could have presented our readers with each author's opinion of the merits of the work which he did not write.

